# Social Cognition, Executive Functions and Self-Report of Psychological Distress in Huntington’s Disease

**DOI:** 10.1371/currents.hd.bba3a680813122013e6d3e8a144c1da8

**Published:** 2016-12-28

**Authors:** Ida Unmack Larsen, Tua Vinther-Jensen, Jørgen Erik Nielsen, Anders Gade, Asmus Vogel

**Affiliations:** Neurogenetics Clinic, Danish Dementia Research Centre, Department of Neurology, Rigshospitalet, University of Copenhagen, Copenhagen, Denmark; Denmark Department of Psychology, University of Copenhagen, Copenhagen, Denmark; Neurogenetics Clinic, Danish Dementia Research Centre, Department of Neurology, Rigshospitalet, University of Copenhagen, Copenhagen, Denmark; Department of Cellular and Molecular Medicine, Section of Neurogenetics, University of Copenhagen, Copenhagen, Denmark; Neurogenetics Clinic, Danish Dementia Research Centre, Department of Neurology, Rigshospitalet, University of Copenhagen, Copenhagen, Denmark; Department of Cellular and Molecular Medicine, Section of Neurogenetics, University of Copenhagen, Copenhagen, Denmark; Denmark Department of Psychology, University of Copenhagen, Copenhagen, Denmark; Neurogenetics Clinic, Danish Dementia Research Centre, Department of Neurology, Rigshospitalet, University of Copenhagen, Copenhagen, Denmark; Denmark Department of Psychology, University of Copenhagen, Copenhagen, Denmark

**Keywords:** executive functions, Huntington's disease, psychological distress, self-report, social cognition

## Abstract

Objective: Huntington’s disease (HD) is characterized by motor symptoms, psychiatric symptoms and cognitive impairment in, inter alia, executive functions and social cognition. The aim of this study was to investigate the relationship between subjective feeling of psychological distress using a self-report questionnaire and performances on tests of executive functions and social cognition in a large consecutive cohort of HD patients.

Method: 50 manifest HD patients were tested in social cognition and executive functions and each answered a self-report questionnaire about current status of perceived psychological distress (the Symptom Checklist-90-Revised (SCL-90-R)). Correlation analyses of test performance and SCL-90-R scores were made as well as stepwise linear regression analyses with the SCL-90-R GSI score and test performances as dependent variables.

Results: We found that less psychological distress was significantly associated with worse performances on social cognitive tests (mean absolute correlation .34) and that there were no significant correlations between perceived psychological distress and performance on tests of executive functions. The correlations between perceived psychological distress and performance on social cognitive tests remained significant after controlling for age, Unified Huntington’s Disease Rating Scale-99 total motor score and performance on tests of executive functions.

Conclusions: Based on previous findings that insight and apathy are closely connected and may be mediated by overlapping neuroanatomical networks involving the prefrontal cortex and frontostriatal circuits, we speculate that apathy/and or impaired insight may offer an explanation for the correlation between self-report of psychological distress and performance on social cognitive tests in this study.

## Introduction

Huntington’s disease (HD) is an autosomal dominantly inherited neurodegenerative disorder caused by an expanded CAG repeat on chromosome 4^1^ and characterized by motor symptoms, psychiatric symptoms and cognitive decline. Once onset of motor symptoms has occurred, cognitive and/or psychiatric symptoms also tend to be present 2 ^,^ 3 ^,^ 4 ^,^ 5. The cognitive deterioration in HD is thought to be related to dysfunction of the frontostriatal circuits due to gradual degeneration of the striatum^6^^,^ 7. Accordingly, the first signs of cognitive impairment are observed in cognitive functions associated with the prefrontal cortex such as executive functions and social cognitive functions^4^^,^ 8 ^,^ 9 ^,^ 10. Psychiatric symptoms are also commonly found even in the presymptomatic and earliest stages in HD, and a wide variety of psychiatric symptoms such as apathy, depression, irritability, anxiety, mania and obsessive compulsive symptoms can be seen^11^.

Performance on tests of social cognition and executive functions are often affected by psychopathology. Associations between social cognitive functions, executive functions and psychiatric symptoms have been investigated in both psychiatric and neurological disorders such as schizophrenia, depression, bipolar disorder, Parkinson’s disease (PD) and frontotemporal dementia (FTD)^12^^,^ 13 ^,^ 14 ^,^ 15 ^,^ 16 ^,^ 17 ^,^ 18. The most common finding being that greater degree of psychiatric symptoms are associated to worse performance on tests of executive functions and social cognition.

In two previous studies we have investigated performance on tests of executive functions and social cognition in a large cohort of HD patients in early-moderate disease stage and found a high frequency of impaired performances on tests of executive functions and social cognition^2^^,^ 4. Based on the associations between the presence of psychiatric symptoms and impairments in executive functions and social cognition found in other patient groups we wanted to investigate how subjective experience of psychological distress as reported by patients with Huntington’s disease is related to performance on tests of social cognition and executive functions.

Therefore the aim of this study was to investigate the relationship between self-report of perceived psychological distress and performances on a battery of tests of executive functions and social cognition in a large consecutive cohort of HD patients. We wanted to investigate: 1) whether performances on tests of social cognition and executive functions are correlated, and 2) whether performances on tests of social cognition and executive functions are associated with degree of self-reported psychological distress. To our knowledge, this is the first study to compare performance on a large battery of tests of social cognition and executive functions to self-report of psychological distress in HD.

## Methods

**Participants**

Participants were recruited from January 2012 to March 2013 from the Neurogenetics Clinic, Danish Dementia Research Centre, Rigshospitalet. Fifty HD patients with a CAG repeat ≥39, a Unified Huntington’s Disease Rating Scale-99 total motor score (UHDRS-TMS) of >5^19^, a Mini-Mental State Examination (MMSE) score ≥ 24, and a Montreal Cognitive Assessment (MoCA)^20^ score ≥ 20 were included in the study. Exclusion criteria were other neurological illness, ongoing alcohol or drug abuse and having a native language other than Danish. Table 1 shows the background information for the HD patients. All patients had gone through genetic counseling and had been informed of their genetic status prior to (and independently from) study enrolment. We have previously published results from the same cohort^2^^,^ 4 ^,^ 5 ^,^ 21 also including premanifest HD carriers, but since we found no impairments in social cognitive functions in our premanifest subjects we did not include them in the present study.

**Figure:**
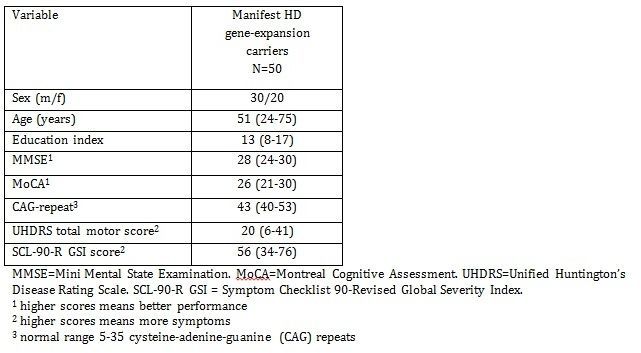
**Table 1.** Background information. Results shown as median (range)

**Procedure**

The study was approved by the Ethics Committee of the Capital Region of Denmark(H2-2011-085), and written informed consent was obtained from each participant before enrollment. All participants had a minimum of two planned visits. At one visit psychiatric screening and neurological examinations were performed. At the other visit neuropsychological testing was performed. The same physician and the same neuropsychologist performed all examinations. The examination by the physician and the examination by the neuropsychologist were performed blinded to one another.

**Neuropsychological Testing**

All participants were tested with an extensive three-hour battery of neuropsychological tests, including tests of attention, memory, visuospatial functions, executive functions and social cognition. For this study only tests of executive functions and tests of social cognition are included. The tests were administered in a fixed order. The education index score (range 8-17) was calculated as the sum of years of schooling (range 7-12) and the level of post-secondary education stratified into groups (range 1-5) based on the method previously used by Mortensen and Gad.^22^

**Emotion Hexagon (EH)** 23: This test consists of 30 cards with pictures of morphed facial expressions of the six basic emotions: happiness, surprise, fear, sadness, anger and disgust. Each of the six emotions was represented with four pictures; each picture was morphed with either 10% or 30% of the neighboring emotions (e.g. happiness is morphed with either 10% or 30% anger or surprise). Between two neighboring emotions was a picture morphed with 50% of each emotion; these were not counted in the total raw score. A card with the six emotion words was presented, and each of the six emotions was explained before the pictures were shown to the participants and remained visible for them during the test. The pictures were shown in random order, and the participants were asked to choose which of the six emotions best described the facial expression. The pictures were shown only once, and no feedback was given. The test was scored as the total number of correct responses (0-24).

**Emotion Evaluation Task (EET)** 24: The EET consists of 28 short videotaped vignettes (15-60 seconds) of actors interacting in everyday situations. In some of the scenes there is one actor only, and in other scenes there are two (the participant was then told on whom to focus). The participants were asked to choose whether the actor was displaying one of the six basic emotions: happiness, surprise, sadness, anger, fear or disgust or no particular emotion (neutral). The EET does not exist in a Danish version and therefore the video clips were shown without sound in order to exclude any influence of differences in English language abilities. Each video was shown once, and no feedback was given. The test was scored as the total number of correct responses (0-28).

**Social Inference - Minimal (SI-M)** 24 ^,^ 51: The SI-M (Danish version: (Bliksted, Fagerlund, Weed, Frith, & Videbech, 2014) consists of short (15-53 seconds) videotaped vignettes with professional actors interacting in everyday situations. The exchanges are either sincere or sarcastic. The sarcastic vignettes are either with simple sarcasm, meaning that they are acted in such a way as to imply the opposite meaning to what is actually being said, or with paradoxical sarcasm meaning that the exchange of words is meaningless unless one understands that one of the actors is being sarcastic. After each video, the participant was asked four yes/no questions about the interaction. Correct answers to the questions for the sarcastic videos required interpretation of paralinguistic cues such as tone of voice and non-verbal cues such as posture and facial expressions. The test comprises part A2 and part B2. Part A2 consists of five videos of paradoxical sarcasm and ten vignettes that are either sincere or with simple sarcasm. Part B2 consists of exactly the same dialogue as the ten sincere or simple sarcastic videos from part A2 but with sincerity and sarcasm switched. For this study the participants were shown all 25 videos. Each video was shown once, and no feedback was given. Total number of correct yes/no answers (0-100) was used in the present study.

**Reading the Mind in the Eyes Test (RME)** 25: The RME revised version consists of 36 photos of eyes expressing different emotional states. The participants were given four choices of words and were asked to pick the word that best described what the eyes expressed (e.g., serious, ashamed, alarmed or bewildered). In order to pick the correct emotion the participant needed to be able to attribute mental states to others thereby using Theory of Mind (ToM). The participants were also given a list of explanations of all the words in the test and were encouraged to look up the words if they felt uncertain of the meaning of a word. The number of correct responses was recorded (0-36).

**Semantic fluency** 26: The participants were asked to name as many different animals as they could think of within one minute. It was emphasized that all types/categories of animals would be correct. Categories (e.g., birds) as well as specific animals (e.g., eagle) were accepted. The number of different animals named was recorded.

**Lexical fluency** 26: The participants were asked to produce as many words as possible within one minute beginning with each of the letters F, A or S. It was emphasized that it could be all words in Danish except proper nouns. The number of different words produced with F, A and S were recorded and added together for a total score.

**Lexical alternating fluency**. This fluency test was developed by the researchers based on the most common first-letters in Danish apart from S and F. The participants were asked to produce as many different words as possible within one minute, alternating between words beginning with the letter K and words beginning with the letter B. It was emphasized that it could be all types of words except proper nouns. The number of correct responses was recorded, and improper alternations were counted as incorrect.

**Semantic/lexical alternating fluency. **This fluency test was developed by the researchers based on a category that was thought to be very broad and one of the most common first-letters in Danish apart from S, F, K and B. The participants were asked to produce as many different words as possible within one minute alternating between types of food and words beginning with the letter D. They were told that the former could be “anything you can eat”, and that the latter covered all words beginning with the letter D except proper nouns. The number of correct responses was recorded; improper alternations were counted as incorrect.

**Trail Making Test B (TMT B)** 27:The participants were asked to connect circles alternating between numbers in numeric order and letters in alphabetical order. The time to completion was recorded.

**Stroop interference test** 28: This 100-word version of the Stroop test consisted of a simple reading task and an interference test. In the interference test the name of the color and the color of the ink did not correspond, for example the word ‘blue’ could be written in green ink, and the participants were asked to name the colors instead of reading the words. Participants were instructed to complete the test as quickly as possible and to correct their mistakes. Only the time to completion for the interference test was used for analysis.

**Self-report of psychological distress**

**Symptom Checklist -90-Revised (SCL-90-R)** 29: The SCL-90-R is a 90 item self-report inventory designed to reflect the current status of perceived psychological distress. The participant is asked to rate each of the 90 items on a five-point Likert scale ranging from “not at all” to “very often” according to how much they experienced each symptom in the preceding week. The scoring is based on nine primary symptom dimensions: somatization, obsessive-compulsive, interpersonal sensitivity, depression, anxiety, hostility, phobic anxiety, paranoid ideation and psychoticism. Added together these yield three global indices of distress: Global Severity Index (GSI), Positive Symptoms Distress Index and Positive Symptom Total. For the current study raw scores were converted into T-scores standardized to a normative Danish sample stratified by gender^50^. Higher T-scores indicate greater degree of psychological distress. Only the GSI score was used for the association analyses in current study.

**Statistical analysis**

Results for the background variables are presented as medians and ranges. Pearson's r and Spearman's Rho (rs used for skewed distributions) were used to assess the level of significance of correlations between the cognitive tests and the SCL-90-R GSI scores and also to investigate the level of significance of associations between performances on tests of social cognition and executive functions. Lastly stepwise linear regression analyses were used with SCL-90-R GSI score as the dependent variable and scores on each of the social cognitive tests that were significantly associated with SCL-90-R GSI score as independent variables. For each regression analysis performances on all of the executive tests (Lexical fluency, Semantic fluency, Lexical alternating fluency, Semantic/lexical alternating fluency, Stroop test and TMT B), age and UHDRS-TMS were also included as independent variables. Plots of residuals were used as model control and the alpha level was set to .05 (two-tailed).

## Results

Table 2 shows the median and interquartile range for performance on all tests of executive functions and social cognition, as well as the median and interquartile range for the standardized T-scores on the GSI and the nine symptom dimensions of the SCL-90-R.

**Figure:**
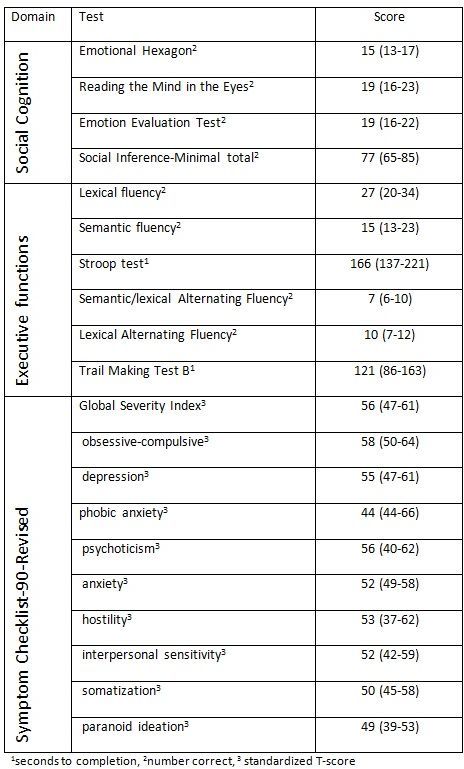
**Table 2.** Scores for HD carriers on the cognitive tests and the Symptom Checklist-90-Revised Global Severity index and the nine primary symptoms dimensions. Results shown as median (interquartile range)

Table 3 shows the correlations between social cognitive tests and tests of executive functions. As expected we found that performances on all four social cognitive tests were significantly correlated with each other. Performances on all four social cognitive tests were also significantly associated with performances on tests of executive functions (mean correlation .24).

**Figure:**
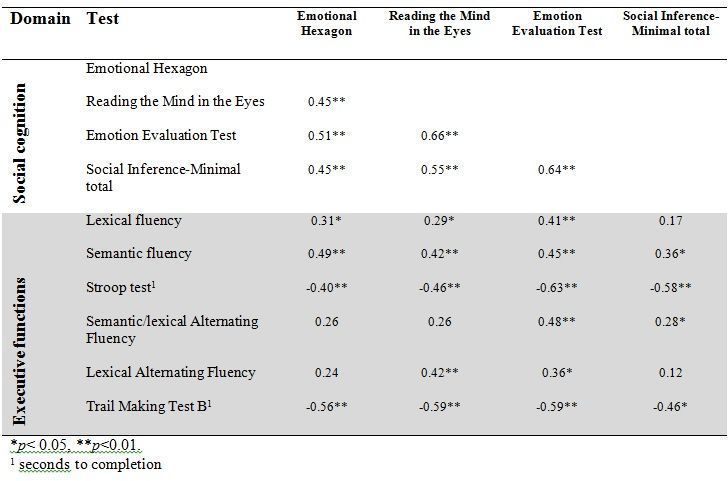
**Table 3.** Correlations between tests of executive functions and tests of social cognition. Results shown as Spearmans Rho.

Table 4 shows the correlations between the SCL-90-R GSI score and the UHDRS-TMS and the tests of executive functions and social cognition. We found a significant correlation between the SCL-90-R GSI score and scores on three of the four social cognitive tests (the SI-M total, the EET and the RME), such that better test performance was related to more perceived psychological distress. The mean overall correlation was .34. By contrast, none of the correlation coefficients between the SCL-90-R GSI score and the tests of executive functions reached significance. Motor symptoms were not significantly correlated to SCL-90-R GSI score, but were significantly correlated to performance on most tests of executive functions and social cognition.

**Figure:**
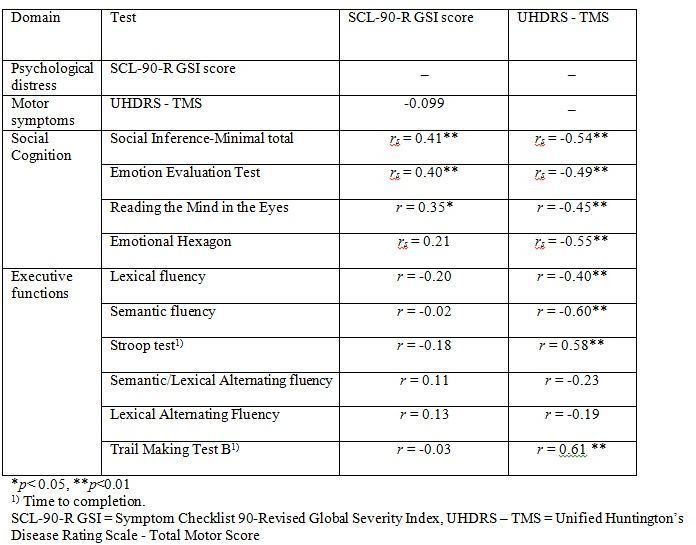
**Table 4.** Correlations between SCL-90-R GSI score, UHDRS – TMS and test of executive functions, social cognition. Results shown as Pearsons r (r) or Spearmans Rho (rs).

Table 5 shows the result from the stepwise linear regression analysis. We found that the correlation between score on SCL-90-R GSI and RME, EET and SI-M score remained significant after including performances on all of the executive function tests (Lexical fluency, Semantic fluency, Lexical alternating fluency, Semantic/lexical alternating fluency, Stroop test and TMT B), age and UHDRS-TMS in the analysis. We also found a significant negative effect of age on SCL-90-R GSI score.

**Figure:**
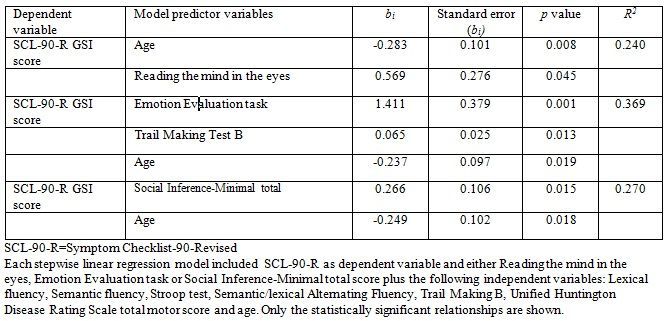
**Table 5.** Stepwise linear regression results

## Discussion

This study investigated whether performances on tests of social cognition and executive functions were associated with perceived psychological distress in a group of HD patients. Since greater degree of psychiatric symptoms such as depression has been negatively associated with both social cognitive skills and executive functions, we were surprised that more perceived psychological distress was significantly associated with better performances on social cognitive tests and that less perceived psychological distress was associated with worse performance on social cognitive tests. The correlations between psychological distress and tests of executive functions were non-significant. Furthermore, the correlations between self-reported psychological distress and performance on social cognitive tests does not seem to be an artefact of those being furthest in the disease progression performing worse and reporting fewer psychological symptoms since the associations remained significant after controlling for age, UHDRS-TMS and performance on tests of executive functions. This means that for our cohort of HD patients feeling less psychologically distressed themselves was associated to worse performance on tests of the ability to recognize emotions, ToM and sarcasm in others. This is an interesting finding that may help to understand the interpersonal problems often associated with HD. It may be helpful for clinicians and caregivers to know that feeling little psychological distress oneself may also influence the ability to recognize distress in others.

We used a self-report measure of psychological distress, and it is important to remember that self-report of psychological distress in HD may be influenced by poor insight, which is a common feature of HD^30^^,^ 31. This means that although the patients themselves do not report having psychiatric symptoms this might not be true from a clinician or caregiver perspective. This means that our findings do not necessarily indicate that poor social cognition is genuinely associated with fewer psychiatric symptoms in patients with HD from an outside perspective. Instead, our findings may reflect a relationship between insight and emotion recognition as has been demonstrated in related disorders^32^ and a close connection between our understanding of the self and others^33^.

Social developmental research has long posited the idea that representations of the self and other are closely connected^34^, and research in neuroscience has supported the view of common representation networks between self and other involving, in particular, the parietal cortex, superior temporal sulcus, limbic areas, striatum and orbital and medial areas in the prefrontal cortex^33^^,^ 35 ^,^ 36 ^,^ 37. Since representations of others are closely connected to our representation of the self, it could be speculated that loss of insight and flattening of affect in oneself may be associated with less sensitivity to others’ emotional states as well, leading to a state of being ‘comfortably numb’. We speculate that an alternative explanation of our results may be that a flattening of affect (from here on referred to as ‘apathy’) and impaired insight has led to low self-report of psychological distress in our cohort and that this was related to the weaker understanding of others (i.e. poor social cognitive skills including emotion recognition).

Impaired insight and apathy are well-known features of HD^31^^,^ 38 ^,^ 39 and other psychiatric and neurological disorders affecting the prefrontal cortex and frontostriatal circuits such as schizophrenia, PD and FTD. Apathy ratings have been associated with white matter changes in the orbitofrontal cortex in HD, and in both PD and FTD apathy has been associated with areas of the striatum and frontostriatal circuits^13^^,^ 17. In schizophrenia negative symptoms have been inversely associated with striatal activation^40^. These studies suggest that social cognition, insight and apathy may in part be mediated by overlapping neuroanatomical networks involving the prefrontal cortex and frontostriatal circuits. Thus HD may be a relevant condition in which such associations can be studied.

Studies of patients with HD, schizophrenia and FTD suggest a reciprocal relationship between insight and apathy (or negative symptoms), such that higher scores of apathy or negative symptoms have been associated with impaired awareness of these symptoms^30^^,^ 41 ^,^ 42. In fact, impaired insight has been associated with better mood in both HD and schizophrenia meaning that patients with less awareness of illness and symptoms has been associated with fewer symptoms of depression, whereas greater self-awareness has been associated with higher depression scores^14^^,^ 42 ^,^ 43 ^,^ 44. These findings support the view that apathy and lack of insight are closely connected and they also point in the direction of patients with symptoms of apathy being expected also to have poor self-awareness and therefore to report neither apathetic symptoms nor psychological distress in general.

Impaired insight and higher apathy scores have been found to be associated with worse performances on social cognitive tests in FTD, PD and schizophrenia^13^^,^ 41 ^,^ 42 ^,^ 45 ^,^ 46. One study in FTD found that patients’ own ratings of apathy were positively correlated to ToM whereas the caregivers’ apathy ratings were negatively associated with ToM performance^13^, indicating that greater awareness of apathetic symptoms was associated with better social cognitive skills. This finding may reflect the close connection between representations of the self and other and is in line with our results indicating that patients with better self-awareness also have a better understanding of others’ emotional states and vice versa. We found no associations between self-report of psychological distress and performance on tests of executive functions. This was somewhat surprising since psychiatric symptoms such as depression has often been associated to impairment in executive functions. There may be several different explanations for this finding. It may be that the tests used in this study are not sensitive to the type of executive dysfunction associated with psychiatric symptoms. It could also be that the types of psychiatric symptoms that are associated with executive dysfunctions are not well measured by the SCL-90-R. In a study by Thompson et al. 47 they found that only apathy was significantly associated with impairment in executive functions in HD.

No other studies that we know of have found significant correlations between self-report of psychological distress and performance on social cognitive tests in HD. Two studies have investigated the correlation between alexithymia and emotion recognition and ToM in HD but found no significant correlations^48^, and one other study found no significant associations between performance on a ToM task and psychiatric symptoms on the Positive And Negative Syndrome Scale in HD^49^. Discrepancies between our results and previous findings may be explained by different methodological approaches, and these previous studies had small sample sizes compared to our study.

**Limitations**

There are several limitations to our study. In an exploratory study like this was, there can be quite a lot of statistical comparisons and thus a chance of false positive findings. The use of a self-report measure of perceived psychological distress may have been misleading, since some HD patients are known to show poor awareness of their symptoms. Future research should add ratings from caregivers or clinicians to overcome this problem and to get more information about the relationship between social cognitive performances and insight in HD. Also an apathy rating scale or a scale for rating negative symptoms, would have helped to clarify the mechanisms for our findings. Future studies should apply apathy ratings to investigate the association between social cognition and apathy in HD.

Other limitations to the study relate to the tests used. The Danish version of TASIT was developed for research purposes and it has not been standardized and validated. This must, of course, lead to caution when interpreting results. Sarcasm as a part of everyday interaction is somewhat culture specific and thus generalization to all other cultures may be limited. The EET of the TASIT does not exist in a Danish version and thus was used without sound. The clinical impression of performances on this test was that it was still meaningful and there were no significant differences between healthy controls and premanifest HD carriers on performances on the EET in a previously published study (ref). Although our evaluation was that the test was still meaningful, the different use of the test from its original form requires caution when interpreting the results.

## Conclusions

We found significant associations between self-report of psychological distress and performances on social cognitive tests but not on tests of executive functions. According to our findings HD patients that feel less psychologically distressed themselves perform worse on tests of the ability to recognize emotions, ToM and sarcasm in others. This is an interesting finding that may be of importance for understanding the interpersonal problems often associated with HD. We speculate that one mechanism for this finding may be that shared representations of self and other as well as insight and apathy may be closely connected and may be mediated by overlapping neuroanatomical networks involving the prefrontal cortex and frontostriatal circuits. Therefore we speculate that a flattening of affect or apathy and impaired insight has led to low self-report of psychological distress in our cohort and that this was related to the weaker understanding of others (i.e. poor social cognitive skills including emotion recognition).

## Competing Interests Statement

The authors have declared that no competing interests exist

## Data Availability Statement

All relevant data are within the manuscript.

## Corresponding Author

Ida Unmack Larsen (ida.unmack.larsen.01@regionh.dk)

## References

[1] A novel gene containing a trinucleotide repeat that is expanded and unstable on Huntington's disease chromosomes. The Huntington's Disease Collaborative Research Group. Cell. 1993 Mar 26;72(6):971-83. PubMed PMID:8458085. 8458085 10.1016/0092-8674(93)90585-e

[2] Larsen IU, Vinther-Jensen T, Gade A, Nielsen JE, Vogel A. Do I misconstrue? Sarcasm detection, emotion recognition, and theory of mind in Huntington disease. Neuropsychology. 2016 Feb;30(2):181-9. PubMed PMID:26301773. 26301773 10.1037/neu0000224

[3] Paulsen JS, Ready RE, Hamilton JM, Mega MS, Cummings JL. Neuropsychiatric aspects of Huntington's disease. J Neurol Neurosurg Psychiatry. 2001 Sep;71(3):310-4. PubMed PMID:11511702. 11511702 10.1136/jnnp.71.3.310 PMC1737562

[4] Unmack Larsen I, Vinther-Jensen T, Gade A, Nielsen JE, Vogel A. Assessing impairment of executive function and psychomotor speed in premanifest and manifest Huntington's disease gene-expansion carriers. J Int Neuropsychol Soc. 2015 Mar;21(3):193-202. PubMed PMID:25850430. 25850430 10.1017/S1355617715000090

[5] Vinther-Jensen T, Larsen IU, Hjermind LE, Budtz-Jørgensen E, Nielsen TT, Nørremølle A, Nielsen JE, Vogel A. A clinical classification acknowledging neuropsychiatric and cognitive impairment in Huntington's disease. Orphanet J Rare Dis. 2014 Jul 17;9:114. PubMed PMID:25026978. 25026978 10.1186/s13023-014-0114-8 PMC4105878

[6] Alexander GE, DeLong MR, Strick PL. Parallel organization of functionally segregated circuits linking basal ganglia and cortex. Annu Rev Neurosci. 1986;9:357-81. PubMed PMID:3085570. 3085570 10.1146/annurev.ne.09.030186.002041

[7] Tabrizi SJ, Scahill RI, Durr A, Roos RA, Leavitt BR, Jones R, Landwehrmeyer GB, Fox NC, Johnson H, Hicks SL, Kennard C, Craufurd D, Frost C, Langbehn DR, Reilmann R, Stout JC. Biological and clinical changes in premanifest and early stage Huntington's disease in the TRACK-HD study: the 12-month longitudinal analysis. Lancet Neurol. 2011 Jan;10(1):31-42. PubMed PMID:21130037. 21130037 10.1016/S1474-4422(10)70276-3

[8] Dumas EM, van den Bogaard SJ, Middelkoop HA, Roos RA. A review of cognition in Huntington's disease. Front Biosci (Schol Ed). 2013 Jan 1;5:1-18. PubMed PMID:23277034. 23277034 10.2741/s355

[9] Eddy CM, Rickards HE. Theory of mind can be impaired prior to motor onset in Huntington's disease. Neuropsychology. 2015 Sep;29(5):792-8. PubMed PMID:25664466. 25664466 10.1037/neu0000190

[10] Stout JC, Paulsen JS, Queller S, Solomon AC, Whitlock KB, Campbell JC, Carlozzi N, Duff K, Beglinger LJ, Langbehn DR, Johnson SA, Biglan KM, Aylward EH. Neurocognitive signs in prodromal Huntington disease. Neuropsychology. 2011 Jan;25(1):1-14. PubMed PMID:20919768. 20919768 10.1037/a0020937 PMC3017660

[11] Craufurd D, Snowden JS. Neuropsychiatry and Neuropsychology. In: Bates G, Tabrizi SJ, Jones L, eds. Huntington's Disease, 4 ed. New Yourk: Oxford University Press, 2014:36-65.

[12] Blumberg HP, Stern E, Ricketts S, Martinez D, de Asis J, White T, Epstein J, Isenberg N, McBride PA, Kemperman I, Emmerich S, Dhawan V, Eidelberg D, Kocsis JH, Silbersweig DA. Rostral and orbital prefrontal cortex dysfunction in the manic state of bipolar disorder. Am J Psychiatry. 1999 Dec;156(12):1986-8. PubMed PMID:10588416. 10588416 10.1176/ajp.156.12.1986

[13] Eslinger PJ, Moore P, Antani S, Anderson C, Grossman M. Apathy in frontotemporal dementia: behavioral and neuroimaging correlates. Behavioural neurology 2012;25:127-136. 10.3233/BEN-2011-0351 PMC3640327 22425723

[14] Hoertnagl CM, Hofer A. Social cognition in serious mental illness. Curr Opin Psychiatry 2014 May;27:197-202. 10.1097/YCO.0000000000000055 24613983

[15] Hoff AL, Kremen WS. Is there a cognitive phenotype for schizophrenia: the nature and course of the disturbance in cognition? Current opinion in psychiatry 2002;15:43-48.

[16] Lam BY, Raine A, Lee TM. The relationship between neurocognition and symptomatology in people with schizophrenia: social cognition as the mediator. BMC psychiatry 2014;14:138. 10.1186/1471-244X-14-138 PMC4026589 24885177

[17] Pagonabarraga J, Kulisevsky J, Strafella AP, Krack P. Apathy in Parkinson's disease: clinical features, neural substrates, diagnosis, and treatment. Lancet Neurol 2015;14:518-531. 10.1016/S1474-4422(15)00019-8 25895932

[18] Porter RJ, Bourke C, Gallagher P. Neuropsychological impairment in major depression: its nature, origin and clinical significance. Aust N Z J Psychiatry 2007 Feb;41:115-128. 10.1080/00048670601109881 17464689

[19] Unified Huntington's Disease Rating Scale: reliability and consistency. Huntington Study Group. Mov Disord 1996 Mar;11:136-142. 10.1002/mds.870110204 8684382

[20] Nasreddine ZS, Phillips NA, Bedirian V, et al. The Montreal Cognitive Assessment, MoCA: a brief screening tool for mild cognitive impairment. J Am Geriatr Soc 2005 Apr;53:695-699. 10.1111/j.1532-5415.2005.53221.x 15817019

[21] Vinther-Jensen T, Nielsen TT, Budtz-Jørgensen E, et al. Psychiatric and cognitive symptoms in Huntington's disease are modified by polymorphisms in catecholamine regulating enzyme genes. Clinical genetics 2015;89:320-327. 10.1111/cge.12628 26081309

[22] Mortensen EL, Gade A. On the relation between demographic variables and neuropsychological test performance. Scandinavian Journal of Psychology 1993;34:305-317.

[23] Sprengelmeyer R, Young AW, Calder AJ, et al. Loss of disgust. Perception of faces and emotions in Huntington's disease. Brain 1996 Oct;119 ( Pt 5):1647-1665. 10.1093/brain/119.5.1647 8931587

[24] McDonald S, Flanagan S, Rollins J, Kinch J. TASIT: A new clinical tool for assessing social perception after traumatic brain injury. J Head Trauma Rehabil 2003 May;18:219-238. 10.1097/00001199-200305000-00001 12802165

[25] Baron-Cohen S, Wheelwright S, Hill J, Raste Y, Plumb I. The "Reading the Mind in the Eyes" Test revised version: a study with normal adults, and adults with Asperger syndrome or high-functioning autism. J Child Psychol Psychiatry 2001 Feb;42:241-251. 11280420

[26] Lezak MD, Howieson DB, Loring DW. Executive Functions and Motor Performance. Neuropsychological assessment, fourth ed. New York: Oxford University Press, 2004:611-646.

[27] Reitan RM. The relation of the trail making test to organic brain damage. J Consult Psychol 1955;19:393-394. 10.1037/h0044509 13263471

[28] Stroop JR. Studies of interference in serial verbal reactions. Journal of Experimental Psychology 1935;18:643-662.

[29] Derogatis LR. SCL-90-R Symptom Checklist 90-R. Copenhagen: Psykologisk Forlag A/S, 1977.

[30] Chatterjee A, Anderson KE, Moskowitz CB, Hauser WA, Marder KS. A comparison of self-report and caregiver assessment of depression, apathy, and irritability in Huntington's disease. The Journal of neuropsychiatry and clinical neurosciences 2014;17:378-383. 10.1176/jnp.17.3.378 16179661

[31] Sitek EJ, Thompson JC, Craufurd D, Snowden JS. Unawareness of Deficits in Huntington's Disease. Journal of Huntington's disease 2014;3:125-135. 10.3233/JHD-140109 25062855

[32] O'Keeffe FM, Murray B, Coen RF, et al. Loss of insight in frontotemporal dementia, corticobasal degeneration and progressive supranuclear palsy. Brain 2007;130:753-764. 10.1093/brain/awl367 17347257

[33] Decety J, Sommerville JA. Shared representations between self and other: a social cognitive neuroscience view. Trends in cognitive sciences 2003;7:527-533. 10.1016/j.tics.2003.10.004 14643368

[34] Gallagher S, Meltzoff AN. The earliest sense of self and others: Merleau-Ponty and recent developmental studies. Philosophical psychology 1996;9:211-233. 10.1080/09515089608573181 PMC3845406 24307757

[35] Abu-Akel A. A neurobiological mapping of theory of mind. Brain research reviews 2003;43:29-40. 10.1016/s0165-0173(03)00190-5 14499460

[36] Abu-Akel A, Shamay-Tsoory S. Neuroanatomical and neurochemical bases of theory of mind. Neuropsychologia 2011;49:2971-2984. 10.1016/j.neuropsychologia.2011.07.012 21803062

[37] Lieberman MD. Social cognitive neuroscience: a review of core processes. Annual Review of Psychology 2007;58:259-289. 10.1146/annurev.psych.58.110405.085654 17002553

[38] Tabrizi SJ, Scahill RI, Owen G, et al. Predictors of phenotypic progression and disease onset in premanifest and early-stage Huntington's disease in the TRACK-HD study: analysis of 36-month observational data. Lancet Neurol 2013 Jul;12:637-649. 10.1016/S1474-4422(13)70088-7 23664844

[39] Thompson JC, Harris J, Sollom AC, et al. Longitudinal evaluation of neuropsychiatric symptoms in Huntington's disease. Journal of Neuropsychiatry and Clinical Neuroscience 2012;24:53-60. 10.1176/appi.neuropsych.11030057 22450614

[40] Ehrlich S, Yendiki A, Greve DN, et al. Striatal function in relation to negative symptoms in schizophrenia. Psychological medicine 2012;42:267-282. 10.1017/S003329171100119X 21733291

[41] Eslinger PJ, Dennis K, Moore P, Antani S, Hauck R, Grossman M. Metacognitive deficits in frontotemporal dementia. Journal of Neurology, Neurosurgery & Psychiatry 2005;76:1630-1635. 10.1136/jnnp.2004.053157 PMC1739430 16291884

[42] Konstantakopoulos G, Ploumpidis D, Oulis P, et al. The relationship between insight and theory of mind in schizophrenia. Schizophrenia research 2014;152:217-222. 10.1016/j.schres.2013.11.022 24321712

[43] Hoth KF, Paulsen JS, Moser DJ, Tranel D, Clark LA, Bechara A. Patients with Huntington's disease have impaired awareness of cognitive, emotional, and functional abilities. J Clin Exp Neuropsychol 2007 May;29:365-376. 10.1080/13803390600718958 17497560

[44] McCusker EA, Gunn DG, Epping EA, et al. Unawareness of motor phenoconversion in Huntington disease. Neurology 2013;81:1141-1147. 10.1212/WNL.0b013e3182a55f05 PMC3795599 23966256

[45] Ng R, Fish S, Granholm E. Insight and theory of mind in schizophrenia. Psychiatry Res. 2015 Jan 30;225(1-2):169-74. PubMed PMID:25467703. 25467703 10.1016/j.psychres.2014.11.010 PMC4269286

[46] Santangelo G, Vitale C, Trojano L, et al. Neuropsychological correlates of theory of mind in patients with early Parkinson's disease. Movement Disorders 2012;27:98-105. 10.1002/mds.23949 21915910

[47] Thompson JC, Snowden JS, Craufurd D, Neary D. Behavior in Huntington's disease: dissociating cognition-based and mood-based changes. J Neuropsychiatry Clin Neurosci 2002;14:37-43. 10.1176/jnp.14.1.37 11884653

[48] Eddy CM, Rickards HE. Interaction without intent: The shape of the social world in Huntington's disease. Social cognitive and affective neuroscience 2015;10:1028-1035. 10.1093/scan/nsv012 PMC4560946 25680992

[49] Brune M, Blank K, Witthaus H, Saft C. "Theory of mind" is impaired in Huntington's disease. Mov Disord 2011 Mar;26:671-678. 10.1002/mds.23494 21384426

[50] Olsen LR, Mortensen EL, Bech P: Mental distress in the Danish general population. Acta Psychiatr Scand 2006, 113:477-484 10.1111/j.1600-0447.2005.00743.x 16677224

[51] The Awareness of Social Inference Test (TASIT): Danish Translation Copyright © (2008) by Skye McDonald, Sharon Flanagan and Jennifer Rollins, published by Pearson Assessment; Copyright © 2011 by Skye McDonald, Sharon Flanagan and Jennifer Rollins. Reproduced with permission from Pearson Assessment. All rights reserved.

